# Supplementary material for: Combination of Coagulation–Flocculation–Decantation and Ozonation Processes for Winery Wastewater Treatment
Source: Int J Environ Res Public Health. 2021 Aug 23;18(16):8882. doi: 10.3390/ijerph18168882 (PMC8395062; doi:10.3390/ijerph18168882)
Supplement: Supplementary file 1 [file ijerph-18-08882-s001.zip › ijerph-1316497-supplementary.pdf]

Supplementary Materials

Combination of Coagulation–Flocculation–Decantation and Ozonation Processes for Winery Wastewater Treatment

Nuno Jorge [1,2], Ana R. Teixeira [2], Carlos C. Matos [2], Marco S. Lucas [2] and José A. Peres [2,*]

[1] Escuela Internacional de Doctorado (EIDO), Campus da Auga, Campus Universitário de Ourense, Universidade de Vigo, As Lagoas, 32004 Ourense, Spain; njorge@uvigo.es

[2] Centro de Química de Vila Real (CQVR), Departamento de Química, Universidade de Trás-os-Montes e Alto Douro (UTAD), Quinta de Prados, 5000-801 Vila Real, Portugal; ritamourateixeira@gmail.com (A.R.T.); cmatos@utad.pt (C.C.M.); mlucas@utad.pt (M.S.L.)

* Correspondence: jperes@utad.pt

Table S1—ANOVA of the regression for turbidity, TSS, COD and TOC. DF - Degrees of freedom; Seq SS - Sum of square; Adj SS - Sum of adjusted squares; Adj MS - Adjusted average squares; F-Value - *Fisher ratio.*

	Turbidity						TSS						COD						TOC					
Source	DF	Seq SS	Adj SS	Adj MS	*F*-Value	*P*-Value	DF	Seq SS	Adj SS	Adj MS	*F*-Value	*P*-Value	DF	Seq SS	Adj SS	Adj MS	*F*-Value	*P*-Value	DF	Seq SS	Adj SS	Adj MS	*F*-Value	*P*-Value
Regression	5	0.41	0.41	0.08	43.55	0.115	5	0.44	0.44	0.09	9.59	0.240	5	34.84	34.84	6.97	4.26	0.352	5	49.72	49.72	9.94	62.03	0.096
Linear regression	2	0.36	0.29	0.14	75.68	0.081	2	0.40	0.32	0.16	17.45	0.167	2	23.34	32.18	16.09	9.83	0.220	2	15.76	37.79	18.89	117.86	0.065
Quadratic regression	3	0.06	0.06	0.02	9.69	0.231	3	0.04	0.04	0.01	1.38	0.542	3	11.50	11.50	3.83	2.34	0.440	3	33.96	33.96	11.32	70.61	0.087
Residual Error	1	0.002	0.002	0.002			1	0.01	0.01	0.01			1	1.64	1.64	1.64			1	0.16	0.16	70.61		
Total	6	0.41					6	0.45					6	36.48					6	49.88				

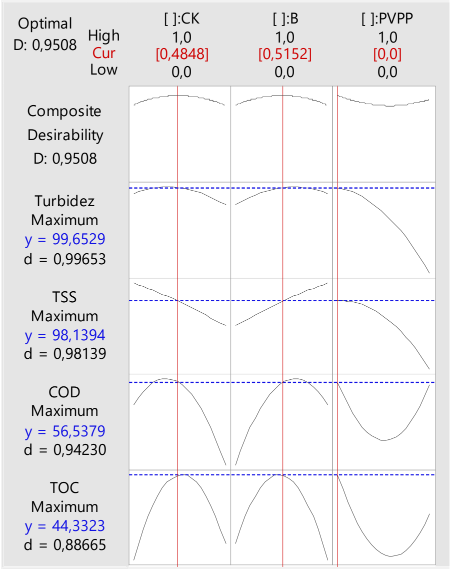

Figure S1 – Optimization chart. Experimental conditions: 0.48 g/L potassium caseinate, 0.52 g/L bentonite, pH 4.0, temperature 298 K, rapid mix 150 rpm/3 min, slow mix 20 rpm/20 min, sedimentation time 12 h. X_1 – Potassium caseinate, X_2 – Bentonite, X_3 – PVPP.

Table S2 – Overall results after coagulation-flocculation-decantation process (CFD). Operational conditions: 0.48 g/L potassium caseinate, 0.52 g/L bentonite, pH 4, temperature 298K, rapid mix 150rpm/3min, slow mix 20rpm/20min, sedimentation time 12h.

Time	Turbidity		TSS		TOC		COD		Total polyphenols	
min	NTU	%	mg/L	%	mg C/L	%	mg O_2/L	%	mg gallic acid/ L	%
0	1040		2430		1962		9432		123	
60	324	68.8	766	68.5	1682	14.3	7794	17.4	38	69.1
120	263	74.7	493	79.7	1450	26.1	7157	24.1	30	75.6
240	106	89.8	284	88.3	1423	27.5	6719	28.8	27	78.0
360	42.3	95.9	127	94.8	1354	31.0	6682	29.2	23	81.3
480	32.1	96.9	91	96.3	1237	37.0	6369	32.5	18	85.4
600	28.8	97.2	83	96.6	1102	43.8	5719	39.4	17	86.2
720	17.6	98.3	58	97.6	1087	44.6	4907	48.0	15	87.8

Table S3 – Evaluation of TOC removal through the ozonation process at different pH values (4.0 – 11). Ozonation experimental conditions: [Fe^{2+}] = 1.0 mM, ozone flow rate 5 mg/min, air flow 1.0 L/min, agitation 350 rpm, time 600min, radiation UV-C mercury lamp (254 nm).

				**O_3/Fe^{2+}/UV-C**				
Time	pH 4.0		pH 7.0		pH 9.0		pH 11.0	
	TOC		TOC		TOC		TOC	
min	mg C/L	%	mg C/L	%	mg C/L	%	mg C/L	%
0	1962		1962		1962		1962	
120	1425	27.4	1870	4.7	1550	21.0	1495	23.8
240	1253	36.1	1403	28.5	1428	27.2	1401	28.6
360	949	51.7	1349	31.2	1343	31.5	1229	37.4
480	801	59.2	1215	38.1	1052	46.4	1101	43.9
600	722	63.2	1131	42.4	927	52.7	988	49.6
				**O_3/UV-C**				
Time	pH 4.0		pH 7.0		pH 9.0		pH 11.0	
	TOC		TOC		TOC		TOC	
min	mg C/L	%	mg C/L	%	mg C/L	%	mg C/L	%
0	1962		1962		1962		1962	
120	1522	22.4	1350	31.2	1386	29.4	1847	5.9
240	1094	44.2	1295	34.0	1386	29.4	1698	13.5
360	1212	38.2	1257	35.9	1308	33.3	1405	28.4
480	1061	45.9	1228	37.4	1246	36.5	1264	35.6
600	834	57.5	1224	37.6	1246	36.5	1217	38.0
				**O_3**				
Time	pH 4.0		pH 7.0		pH 9.0		pH 11.0	
	TOC		TOC		TOC		TOC	
min	mg C/L	%	mg C/L	%	mg C/L	%	mg C/L	%
0	1962		1962		1962		1962	
120	1417	27.8	1610	17.9	1810	7.7	1473	24.9
240	1409	28.2	1542	21.4	1776	9.5	1601	18.4
360	1403	28.5	1542	21.4	1776	9.5	1501	23.5
480	1336	31.9	1537	21.7	1776	9.5	1465	25.3
600	1336	31.9	1400	28.6	1743	11.2	1259	35.8
				**UV-C**				
Time	pH 4.0		pH 7.0		pH 9.0		pH 11.0	
	TOC		TOC		TOC		TOC	
min	mg C/L	%	mg C/L	%	mg C/L	%	mg C/L	%
0	1962		1962		1962		1962	
120	1338	31.8	1962	0.0	1962	0.0	1962	0.0
240	1338	31.8	1962	0.0	1962	0.0	1881	4.1
360	1338	31.8	1962	0.0	1962	0.0	1881	4.1
480	1313	33.1	1945	0.9	1962	0.0	1881	4.1
600	1313	33.1	1945	0.9	1962	0.0	1881	4.1

Table S4 – Evaluation of TOC removal through the ozonation process at different Fe^{2+} concentrations (0.5 – 2.0 mM). Ozonation experimental conditions: pH = 4.0, ozone flow rate 5 mg/min, air flow 1.0 L/min, agitation 350 rpm, time 600min, radiation UV-C mercury lamp (254 nm).

Time	UV-C/Fe^{2+}/O_3						Blanks					
	0.5 mM Fe^{2+}		1.0 mM Fe^{2+}		2.0 mM Fe^{2+}		O_3/UV-C		O_3		UV-C	
	TOC		TOC		TOC		TOC		TOC		TOC	
min	mg C/L	%	mg C/L	%	mg C/L	%	mg C/L	%	mg C/L	%	mg C/L	%
0	1962		1962		1962		1962		1962		1962	
120	1241	36.7	1425	27.4	1258	35.9	1522	22.4	1417	27.8	1338	31.8
240	1061	45.9	1253	36.1	1038	47.1	1094	44.2	1409	28.2	1338	31.8
360	962	51.0	949	51.7	827	57.9	1212	38.2	1403	28.5	1338	31.8
480	872	55.5	801	59.2	760	61.3	1061	45.9	1336	31.9	1313	33.1
600	804	59.0	722	63.2	654	66.7	834	57.5	1336	31.9	1313	33.1

Table S5 – Determination of ozone consumption throughout the ozonation process. Ozonation experimental conditions: pH = 4.0, [Fe^{2+}] = 1.0 mM, ozone flow rate 5 mg/min, air flow 1.0 L/min, agitation 350 rpm, time 600 min, radiation UV-C mercury lamp (254 nm).

Time	Injected ozone	Dissolved ozone	Ozone lost
min	mg O_3/min	mg O_3/min	mg O_3/min
0	5.000	0.000	0.000
120	5.000	0.165	4.835
240	5.000	0.330	4.670
360	5.000	0.190	4.810
480	5.000	0.270	4.730
600	5.000	0.360	4.640

Table S6 – Overall results after ozonation (O_3), coagulation-flocculation-decantation (CFD) and combined O_3/CFD and CFD/O_3 treatments. CFD experimental conditions: 0.48 g/L potassium caseinate, 0.52 g/L bentonite, pH 4, temperature 298 K, rapid mix 150 rpm/3 min, slow mix 20 rpm/20 min, sedimentation time 12h. Ozonation experimental conditions: pH 4.0, [Fe^{2+}] = 1.0 mM, ozone flow rate 5 mg/min, air flow 1.0 L/min, agitation 350 rpm, time 600 min, radiation UV-C mercury lamp (254 nm).

Process		TOC		COD		BOD_5		BOD_5/COD	Turbidity	Removal	TSS	Removal	Total polyphenols	Removal
		mg C/L	%	mg O_2/L	%	mg O_2/L	%		NTU	%	mg/L	%	mg/L	%
	Blanc	1962		9432		2611		0.28	1040		2430		123	
Process 1	O_3	722	63.2	4419	53.1	1265	51.6	0.29	359	65.5	807	66.8	6.0	95.1
	O_3/CFD	665	66.1	3169	66.4	909	65.2	0.29	1.2	99.9	41	98.3	5.0	95.9
Process 2	CFD	1087	44.6	4907	48.0	1580	39.5	0.32	17.6	98.3	58	97.6	23.1	81.2
	CFD/O_3	676	65.5	3707	60.7	1500	42.5	0.40	7.2	99.3	41	98.3	0.9	99.3

Table S7 – Analysis of seed phytotoxicity after wastewater treatment, by evaluation of germination percentage (G), relative seed germination (RSG), relative root growth (RRG) and germination index (GI). CFD experimental conditions: 0.48 g/L potassium caseinate, 0.52 g/L bentonite, pH 4.0, temperature 298 K, rapid mix 150 rpm/3 min, slow mix 20 rpm/20 min, sedimentation time 12h. Ozonation experimental conditions: pH 4.0, [Fe^{2+}] = 1.0 mM, ozone flow rate 5 mg/min, air flow 1.0 L/min, agitation 350 rpm, time 600 min, radiation UV-C mercury lamp (254 nm). GI ≤50% (high concentration of phytotoxic substances), 80%<GI>50% (moderated presence of phytotoxic substances), GI ≥80% (there are no phytotoxic substances, or they exist in very small dosages).

Processes	Treatments	Seed	Root length<br>mm	G<br>%	RSG<br>%	RRG<br>%	GI<br>%
	Pure water	Onion	8.73	69			
		Cucumber	45.85	97			
		Lettuce	3.80	51			
		Corn	74.32	80			
	Raw WW	Onion	12.66	82	119	145	173
		Cucumber	32.50	70	72	71	51
		Lettuce	0.00	0	0	0	0
		Corn	116.94	93	117	157	184
Process 1	O_3	Onion	13.88	91	132	159	210
		Cucumber	57.88	87	90	126	113
		Lettuce	13.03	44	87	343	298
		Corn	68.37	100	125	92	115
	O_3/CFD	Onion	6.65	24	35	76	27
		Cucumber	58.01	83	86	127	109
		Lettuce	5.13	33	65	135	88
		Corn	212.22	100	125	286	357
Process 2	CFD	Onion	7.95	71	103	91	94
		Cucumber	34.86	90	93	76	71
		Lettuce	0.00	0	0	0	0
		Corn	100.04	93	117	135	157
	CFD/O_3	Onion	10.40	60	87	119	104
		Cucumber	59.83	87	90	130	117
		Lettuce	10.37	47	91	273	249
		Corn	89.63	87	108	121	131

Table S8 – Analysis of phenolic composition after ozonation (O_3), coagulation-flocculation-decantation (CFD) and combined O_3/CFD and CFD/O_3 treatments. CFD experimental conditions: 0.48 g/L potassium caseinate, 0.52 g/L bentonite, pH 4.0, temperature 298 K, rapid mix 150 rpm/3 min, slow mix 20 rpm/20 min, sedimentation time 12 h. Ozonation experimental conditions: pH 4.0, [Fe^{2+}] = 1.0 mM, ozone flow rate 5 mg/min, air flow 1.0 L/min, agitation 350 rpm, time 600 min, radiation UV-C mercury lamp (254 nm).

Process		Color intensity	hue	Total phenols		Non-flavonoids		Flavonoids		Total anthocyanins		Colored anthocyanins		Total pigments		Total tannins	
		u.a.	u.a.	mg gallic acid/ L	% removal	mg gallic acid/ L	% removal	mg gallic acid/ L	% removal	mg/ L	% removal	mg/ L	% removal	mg/ L	% removal	mg/ L	% removal
	Blanc	0.95	1.04	157		115		41		4		0.07		0.45		87.0	
Process 1	O_3	0.37	1.76	157	0.0	115	0.0	41	0.0	2	50.0	0.05	28.6	0.05	88.9	19.3	77.8
	O_3/CFD	0.23	1.54	149	5.1	115	0.0	35	14.6	2	50.0	0.00	100	0.10	77.8	0.0	100
Process 2	CFD	0.28	1.30	154	1.9	112	2.6	41	0.0	3	25.0	0.04	42.9	0.10	77.8	29.0	66.7
	CFD/O_3	0.17	1.89	152	3.2	112	2.6	38	7.3	0	100.0	0.00	100	0.00	100	0.0	100

Table S9 – Analysis of chromatic characteristics (CIELab) after ozonation (O_3), coagulation-flocculation-decantation (CFD) and combined $O_3 \rightarrow$ CFD and CFD $\rightarrow O_3$ treatments. CFD experimental conditions: 0.48 g/L potassium caseinate, 0.52 g/L bentonite, pH 4, temperature 298 K, rapid mix 150 rpm/3 min, slow mix 20 rpm/ 20 min, sedimentation time 12h. Ozonation experimental conditions: pH 4.0, [Fe^{2+}] = 1.0 mM, ozone flow rate 5 mg/min, air flow 1.0 L/min, agitation 350 rpm, time 600 min, radiation UV-C mercury lamp (254 nm).

Process		L*(%)	a*	b*	ΔL*	Δa*	Δb*	ΔE_{ab}*
	Blanc	0.00	1.78	4.11				
Process 1	O_3	21.66	2.80	10.16	21.66	1.02	6.05	22.51
	O_3/CFD	99.67	-0.11	1.02	99.67	-1.89	-3.09	99.74
Process 2	CFD	100.00	-0.05	0.50	100.00	-1.83	-3.61	100.00
	CFD/O_3	100.00	-0.12	0.82	100.00	-1.90	-3.29	100.00
